# *Spirulina maxima* Extract Ameliorates Learning and Memory Impairments via Inhibiting GSK-3β Phosphorylation Induced by Intracerebroventricular Injection of Amyloid-β 1–42 in Mice

**DOI:** 10.3390/ijms18112401

**Published:** 2017-11-13

**Authors:** Eun-Jeong Koh, Kui-Jin Kim, Ji-Hyeon Song, Jia Choi, Hyeon Yong Lee, Do-Hyung Kang, Ho Jin Heo, Boo-Yong Lee

**Affiliations:** 1 Department of Food Science and Biotechnology, College of Life Science, CHA University, Seongnam, Kyonggi 13488, Korea; kej763@naver.com (E.-J.K.); Kuijin.Kim@cha.ac.kr (K.-J.K.); redcross0313@naver.com (J.-H.S.); wldk3176@gmail.com (J.C.); 2 Department of Food Science and Engineering, Seowon University, Cheongju 28674, Korea; hyeonl@kangwon.ac.kr; 3 Jeju International Marine Science Center for Research & Education, Korea Institute of Ocean Science & Technology (KIOST), Jeju 63349, Korea; dohkang@kiost.ac.kr; 4 Division of Applied Life Science, Institute of Agriculture and Life Science, Gyeongsang National University, Jinju 52828, Korea; hjher@gnu.ac.kr

**Keywords:** cognitive impairment, *Spirulina maxima* 70% ethanol extract (SM70EE), amyloid precursor protein (APP) processing, oxidative stress, glycogen synthase kinase-3β (GSK-3β)

## Abstract

*Spirulina maxima*, a microalga containing high levels of protein and many polyphenols, including chlorophyll a and C-phycocyanin, has antioxidant and anti-inflammatory therapeutic effects. However, the mechanisms where by *Spirulina maxima* ameliorates cognitive disorders induced by amyloid-β 1–42 (Aβ_1–42_) are not fully understood. In this study, we investigated whether a 70% ethanol extract of *Spirulina maxima* (SM70EE) ameliorated cognitive impairments induced by an intracerebroventricular injection of Aβ_1–42_ in mice. SM70EE increased the step-through latency time in the passive avoidance test and decreased the escape latency time in the Morris water maze test in Aβ_1–42_-injected mice. SM70EE reduced hippocampal Aβ_1–42_ levels and inhibited amyloid precursor protein processing-associated factors in Aβ_1–42_-injected mice. Additionally, acetylcholinesterase activity was suppressed by SM70EE in Aβ_1–42_-injected mice. Hippocampal glutathione levels were examined to determine the effects of SM70EE on oxidative stress in Aβ_1–42_-injected mice. SM70EE increased the levels of glutathione and its associated factors that were reduced in Aβ_1–42_-injected mice. SM70EE also promoted activation of the brain-derived neurotrophic factor/phosphatidylinositol-3 kinase/serine/threonine protein kinase signaling pathway and inhibited glycogen synthase kinase-3β phosphorylation. These findings suggested that SM70EE ameliorated Aβ_1–42_-induced cognitive impairments by inhibiting the increased phosphorylation of glycogen synthase kinase-3β caused by intracerebroventricular injection of Aβ_1–42_ in mice.

## 1. Introduction

Increased amyloid-β (Aβ) in the brain is associated with cognitive impairment [1]. These events are proceeded by processing of amyloid precursor protein (APP). Cleavage of APP by β-site APP-cleaving enzyme 1 (BACE1) and γ-secretase results in the production and deposition of Aβ in the brain [2]. The major Aβ peptide generated by γ-secretase cleavage is Aβ_1–42_ [3], which is more insoluble and neurotoxic [4,5] and has been more strongly associated with cognitive disorders [6] than the other generated peptides.

Increased Aβ deposition affects acetylcholine (ACh) and acetylcholinesterase (AChE) [7]. The neurotransmitter ACh plays a critical role in enhancement of cognitive function and is hydrolyzed by AChE into choline and acetate [8]. The Aβ_1–42_-induced reduction of acetylcholine ACh and elevation of AChE is also associated with cognitive impairment [9,10].

The accumulation of Aβ_1–42_ also affects oxidative stress in the brain [11] through generation of reactive oxygen species (ROS) in mitochondria. The overproduction of ROS causes mitochondrial dysfunction and leads to oxidative stress [12]. Glutathione (GSH) and antioxidant enzymes, such as glutathione peroxidase (GPx) and glutathione reductase (GR), which regulate mitochondrial ROS, are reduced in an Aβ_1–42_-induced mouse model of memory and learning impairment [13].

Glycogen synthase kinase-3β (GSK-3β) is an enzyme involved in numerous intracellular signaling systems [14]. In the brain, phosphorylation of GSK-3β is accelerated by accumulation of Aβ_1–42_ [15]. This increased GSK-3β phosphorylation leads to neuronal cell death and cognition dysfunction [16]. Phosphorylation of GSK-3β is affected by the phosphoinositide-3 kinase (PI3K) serine/threonine protein kinase (Akt) signaling pathway [17], which is involved in neuronal survival and protection [18]. When neurons are damaged by Aβ, PI3K is phosphorylated. Phosphorylation of PI3K activates Akt phosphorylation, which inhibits phosphorylation of GSK-3β by Aβ_1–42_ to protect neuronal cells in the brain [19]. Activation of the PI3K/Akt signaling pathway is upregulated by brain-derived neurotrophic factor (BDNF), aneurotrophin that plays a key role in synaptic plasticity and neuronal survival [20].

*Spirulina maxima*, a microalga, contains abundant nutrients [21] and is used as a food supplement because of its high protein content [22]. This cyanobacterium is well known for its various healthful effects, including its antioxidant, anti-diabetes [23], and anti-inflammatory properties owing to its vitamin and polyphenol, such as chlorophyll a and C-phycocyanin, content [24]. Recently, we elucidated that 70% ethanol extract of *Spirulina maxima* (SM70EE) has preventive effects on the neurotoxicity of trimethyltin (TMT) or scopolamine in HT-22 cells and mice [25]. Scopolamine and TMT cause neuronal cell death during a short period [26,27]. However, cognitive impairment is caused by the gradual accumulation of amyloid-β over a long period of time in general [2,28]. Therefore, we investigated whether SM70EE ameliorated the learning and memory impairments induced by intracerebroventricular (I.C.V.) injection of Aβ_1–42_ to mice and, if so, whether this effect was mediated through regulating the BDNF/PI3K/Akt signaling pathway and GSK-3β activation.

## 2. Results

### 2.1. SM70EE Ameliorates Learning and Memory Impairments via Inhibiting Aβ Accumulation Induced by I.C.V. Injection of Aβ_1–42_ in Mice

**Figure 1:**
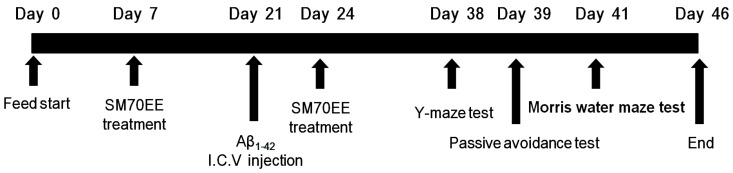
Experimental design. Male institute for cancer research (ICR) mice (5 weeks old) were orally administered a 70% ethanol extract of *Spirulina maxima* (SM70EE; 150 or 450 mg/kg/day) or an equal volume of vehicle (1.0% methylcellulose) once daily for 2 weeks. After 2 weeks, mice received an intracerebroventricular (I.C.V.) injection of Aβ_1–42_ (400 pmol) and continued receiving the same dosage of SM70EE (or vehicle) for an additional 2 weeks. Behavioral tests were conducted after the SM70EE administration concluded.

As shown in Figure 1, SM70EE was orally administered to mice for 2 weeks before and after an I.C.V. injection of Aβ_1–42_, which generates a mouse model of cognitive impairment. To investigate whether SM70EE ameliorated the resulting learning and memory impairments, we conducted passive avoidance and Morris water maze tests with these mice.

**Figure 2:**
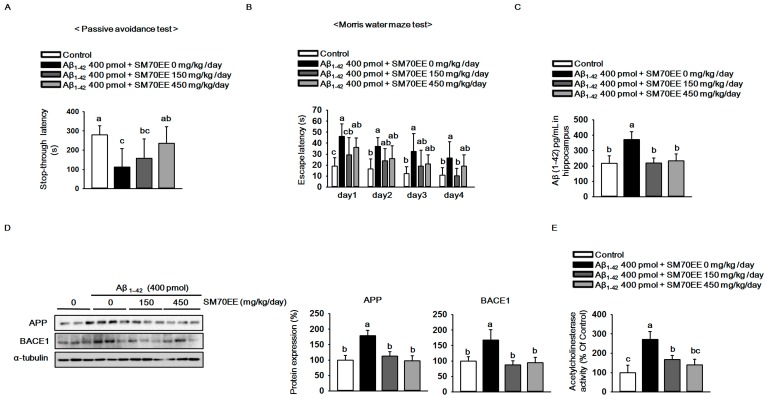
The 70% ethanol extract of *Spirulina maxima* (SM70EE) ameliorates learning and memory impairments by inhibiting the amyloid-β (Aβ) accumulation induced by intracerebroventricular injection of Aβ_1–42_ in mice. (**A**) Mice performed a passive avoidance test 24 h after training (*n* = 6 per group). (**B**) The Morris water maze test was conducted across 4 days (*n* = 6 per group). Behavioral tests results were analyzed by one-way analysis of variance and Duncan’s multiple range test. (**C**) Levels of Aβ_1–42_ in mouse hippocampus (*n* = 4 per group). (**D**) Mouse hippocampal lysates were subjected to western blot analyses to determine amyloid precursor protein (APP) and β-site APP-cleaving enzyme 1 (BACE1) protein expression levels (*n* = 3 per group). Protein expression levels were normalized to α-tubulin. (**E**) Mouse hippocampal lysates were subjected to AChE assay to investigate AChE activity (*n* = 4 per group). Results were analyzed by one-way analysis of variance and Duncan’s multiple range test. The *p*-values in the multiple comparison results (e.g., a, b, c, and d) indicated significant differences among the groups (*p* < 0.05).

As shown in Figure 2A, compared with that for controls, the latency for mice that had received the Aβ_1–42_ I.C.V. injection (400 pmol) to step through to the dark compartment in which they had previously obtained a foot shock was reduced. However, SM70EE dose-dependently increased the step-through latency in the Aβ_1–42_-injected mice. In the Morris water maze task, SM70EE decreased the amount of time Aβ_1–42_-injected mice required to find the submerged platform (Figure 2B). These results showed that SM70EE ameliorated cognitive deficits in mice injected with Aβ_1–42_.

Because APP processing leads to elevated production and accumulation of Aβ in the brain [2] and is accelerated by Aβ_1–42_ injections [29,30], we hypothesized that the SM70EE-induced amelioration of the cognitive impairments observed here were associated with the regulation of APP processing. Thus, we examined the levels of Aβ_1–42_ and APP as well as BACE1 protein, a key factor associated with APP processing, to determine whether SM70EE modulated APP processing. Compared with control mice, those treated with an I.C.V. injection of Aβ_1–42_ (400 pmol) showed increased hippocampal Aβ_1–42_, APP, and BACE1 levels. By contrast, SM70EE administration decreased all of these levels in mouse hippocampus.

Because the AChE activity involved in neurotransmission is also implicated in the function of memory and learning [31] and is elevated by Aβ [32], we next examined the effects of SM70EE on AChE activity. As shown in Figure 2E, SM70EE attenuated the increased AChE activity induced by an I.C.V. Aβ_1–42_ (400 pmol) injection.

These findings indicated that SM70EE ameliorated memory and learning impairment in part by inhibiting the facilitation of Aβ_1–42_ deposition and AChE activity caused by I.C.V. injection of Aβ_1–42_ in mice.

### 2.2. SM70EE Suppresses Hippocampal Oxidative Stress via Upregulating Antioxidant Enzymes Reduced by I.C.V. Injection of Aβ_1–42_

**Figure 3:**
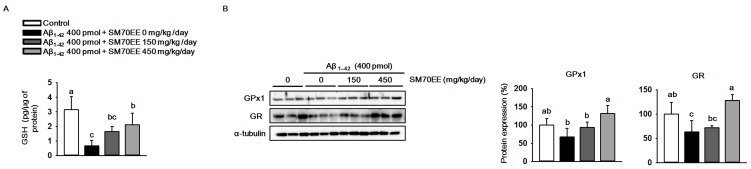
The 70% ethanol extract of *Spirulina maxima* (SM70EE) suppresses oxidative stress by increasing the antioxidant enzyme levels that had been reduced in mouse hippocampus by an intracerebroventricular injection of amyloid-β 1–42 (Aβ_1–42_). (**A**) Glutathione (GSH) levels in mouse hippocampus (*n* = 4 per group). (**B**) Mouse hippocampal lysates were subjected to western blot analyses to determine glutathione peroxidase 1 (GPx1) and glutathione reductase (GR) protein expression levels (*n* = 3 per group). Protein levels were normalized to α-tubulin. Results were analyzed using one-way analysis of variance and Duncan’s multiple range test. The *p*-values in the multiple comparison results (e.g., a, b, c, and d) indicated significant differences among the groups (*p* < 0.05).

Elevation of production and accumulation of Aβ_1–42_ causes mitochondrial dysfunction and results in oxidative stress in the brain [33,34]. To investigate whether SM70EE has inhibitory effects on the oxidative stress induced by the I.C.V. injection of Aβ_1–42_ in mice, we measured the levels of GSH and the antioxidant enzymes GPx1 and GR. As shown in Figure 3A, the level of hippocampal GSH was decreased by the Aβ_1–42_ injection. However, SM70EE administration increased the levels of GSH that had been reduced by Aβ_1–42_ toward control values. Similarly, GPx1 and GR protein expression levels (Figure 3B) were decreased by Aβ_1–42_ and the amount of this decrease was reduced by SM70EE administration. These data indicated that SM70EE attenuated oxidative stress in the hippocampus by enhancing antioxidant systems that had been reduced by I.C.V. injection of Aβ_1–42_ in mice.

### 2.3. SM70EE Inhibits Phosphorylation of GSK-3β in Mouse Hippocampus via Promoting the Activation of the BDNF/PI3K/Akt Signaling Pathways Reduced by I.C.V. Injection of Aβ_1–42_

**Figure 4:**
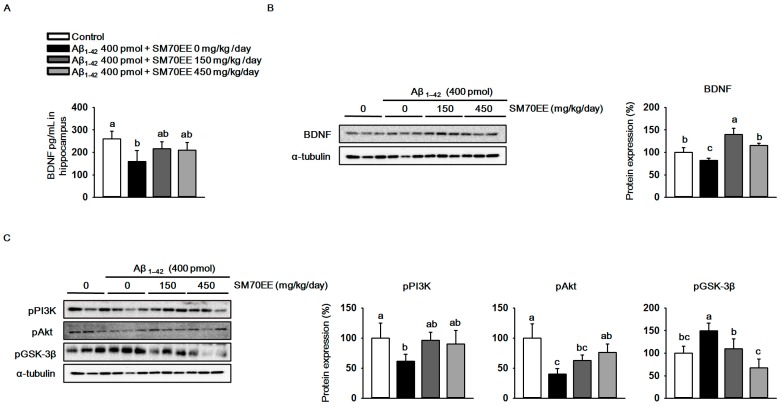
The 70% ethanol extract of *Spirulina maxima* (SM70EE) inhibits amyloid-β 1–42 (Aβ_1–42_)-induced hyperphosphorylation of glycogen synthase kinase-3β (GSK-3β) by activating the Aβ_1–42_-inhibited hippocampal BDNF/PI3K/Akt signaling pathway. (**A**) Levels of brain-derived neurotrophic factor (BDNF) in mouse hippocampus (*n* = 4 per group). (**B**,**C**) Mouse hippocampal lysates were used in western blot analyses to determine protein levels of (**B**) BDNF and (**C**) phosphorylated phosphatidylinositol-3 kinase (pPI3K), phosphorylated serine/threonine protein kinase (pAkt), and phosphorylated glycogen synthase kinase-3β (GSK-3β) (*n* = 3 per group). Protein levels were normalized to α-tubulin. Results were analyzed by one-way analysis of variance and Duncan’s multiple range test. The *p*-values in the multiple comparison results (e.g., a, b, c, and d) indicated significant differences among the groups (*p* < 0.05).

The oxidative stress observed in Aβ_1–42_-induced cognitive disorders decreases BDNF expression [35]. We investigated whether SM70EE increased BDNF levels in mouse hippocampus. As shown in Figure 4A,B, an I.C.V. injection of Aβ_1–42_ (400 pmol) reduced hippocampal BDNF levels and expression. However, SM70EE administration increased the BDNF levels and expression that had been decreased by I.C.V. injection of Aβ_1–42_ (400 pmol).The phosphorylation of GSK-3β is inhibited by the BDNF/PI3K/Akt signaling pathway to protect neurons from cell death induced by Aβ_1–42_ [19]. Thus, we next performed western blot analyses to determine the protein levels of pPI3K, pAkt, and pGSK-3β in mouse hippocampus. We found that administration of SM70EE resulted in the upregulation of pPI3K and pAkt and the suppression of pGSK-3β (Figure 4C). Because the PI3K/Akt signaling pathway is activated by BDNF, our results suggested that SM70EE inhibits phosphorylation of GSK-3β by activating the BDNF/PI3K/Akt signaling pathway in mouse hippocampus to ameliorate the cognitive impairment induced by the I.C.V. injection of Aβ_1–42_.

## 3. Discussion

Memory and learning impairment have been mainly associated with the elevated production and deposition of amyloid-β and tau phosphorylation along with the depletion of acetylcholine receptors and levels [11]. BACE1, a membrane-bound aspartyl protease, plays a critical role in APP processing. APP is cleaved by BACE1 as well as by γ-secretase, and this results in the production and accumulation of Aβ. Previous reports indicate that BACE1 expression levels and activity are elevated in neurons around plaques in the cognitively impaired brain [36,37]. The results of our passive avoidance and Morris water maze tests showed that SM70EE ameliorated the cognitive disorders examined in these tasks in mice that were induced by an I.C.V. injection of Aβ_1–42_. Thus, we hypothesized that this SM70EE-induced amelioration was associated with APP processing.

To test this hypothesis, we examined the levels of Aβ accumulation and APP processing-associated factors. We found that Aβ deposition and the levels of APP and BACE1 were suppressed by SM70EE, suggesting that the SM70EE-induced amelioration of the memory and learning impairments in the behavioral paradigms used here that we observed following I.C.V. Aβ_1–42_ administration were associated with the inhibited APP processing and Aβ accumulation. AChE is another factor associated with cognitive disorders and with Aβ accumulation [38]. AChE interacts with [39] and is increased by [40] Aβ peptides. Thus, the SM70EE-induced inhibition of AChE activity was likely associated with the attenuated production and accumulation of Aβ.

The accumulation of Aβ_1–42_ affects not only cognitive disorders but also oxidative stress [11]. In the brain, ROS are generated and damaged by several factors, such as high levels of catalytic iron and polyunsaturated fatty acids, and relatively low concentrations of antioxidant enzymes [33]. GSH, which is abundant in the brain, is an antioxidant enzyme and reacts with ROS to protect cells. GSH is catalyzed by GPx to reduce H_2_O_2_ to water and form oxidized glutathione (GSSG). The GSSG is recycled back to GSH by GR. Brain GSH levels are decreased in cognitive disorders, whereas GSSG levels are increased [41]. Our data showed that the hippocampal GSH levels reduced by Aβ_1–42_ deposition are elevated by SM70EE administration in the mouse. The observed SM70EE-induced increase in GPx1 and GR expression levels likely resulted in the increased GSH levels. These findings suggested that SM70EE-mediated increases in hippocampal GSH, GPx1, and GR levels inhibited oxidative stress induced by an I.C.V. Aβ_1–42_ injection in mice.

**Figure 5:**
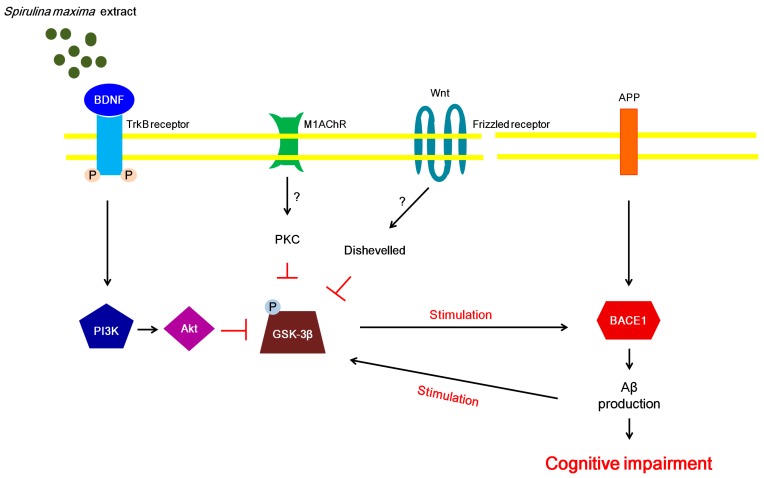
Proposed mechanism whereby SM70EE ameliorates learning and memory impairments in mice with an intracerebroventricular injection of amyloid-β 1–42 (Aβ_1–42_). The 70% ethanol extract of *Spirulina maxima* (SM70EE) inhibits phosphorylation of GSK3-β, which is regulated by the activation of the brain-derived neurotrophic factor (BDNF)/phosphatidylinositol-3 kinase (pPI3K)/serine/threonine protein kinase (Akt) signaling pathway. → indicates stimulation; ⊥, inhibition, ?, unclear action; APP, amyloid precursor protein; BACE1, β-site APP-cleaving enzyme 1; M1AChR, muscarinic acetylcholine receptor M1; PKC, protein kinase C; TrkB, tropomyosin receptor kinase B.

The phosphorylation of GSK-3β is modulated by various signaling pathways, including the Wnt, protein kinase C, and BDNF/PI3K/Akt signaling pathways [19,42]. Recently, it was shown that stimulation of the BDNF/PI3K/Akt signaling pathway leads to the inhibition of GSK-3β phosphorylation [19]. Moreover, activation of the BDNF/PI3K/Akt signaling pathway suppresses oxidative stress caused by Aβ peptides [43]. Thus, we investigated the BDNF/PI3K/Akt signaling pathway to further examine the association between BDNF and oxidative stress induced by Aβ peptides. We speculated that the BDNF levels increased by SM70EE may be associated with the suppression of the oxidative stress that was caused by Aβ accumulation. A recent study demonstrated that GSK-3βphosphorylation regulates BACE1 expression [4]. Thus, we propose that the suppression of hippocampal GSK-3β phosphorylation induced by the SM70EE-enhanced BDNF/PI3K/Akt signaling pathway contributes to the inhibition of APP processing by downregulating BACE1 to ameliorate the cognitive disorders induced by an I.C.V. injection of Aβ_1–42_ (Figure 5).

Taken together, our results suggested that SM70EE attenuates oxidative stress by increasing the Aβ_1–42_-reduced levels of GSH and its associated factors in mice. Moreover, SM70EE ameliorates memory loss and learning impairments through suppression of GSK-3β phosphorylation, which is modulated by BDNF/PI3K/Akt signaling pathway activation.

## 4. Materials and Methods

### 4.1. Materials

SM70EE was provided by the Korea Institute of Ocean Science & Technology (Jeju, Korea) [25]. Phosphate-buffered saline (PBS) was purchased at Gibco (Gaithersburg, MD, USA). Aβ_1–42_ was obtained from Bachem (Bubendorf, Switzerland). Tribromoethanol (Avertin) was purchased from Sigma (St Louis, MO, USA). Hamilton microsyringes (25 μL) were obtained from Hansol Science (Seoul, Korea). The mouse Aβ_1–42_ ELISA kit was purchased from Elabscience (Houston, TX, USA); the GSH assay kit from Cayman Chemical (Ann Arbor, MI, USA); and the mouse BDNF PicoKine ELISA kit from Boster Biological Technology (Pleasanton, CA, USA). The Amplite Colorimetric AChE assay was purchased from AAT Bioquest (Sunnyvale, CA, USA). Unless noted otherwise, all other chemicals were purchased from Sigma.

Antibodies specific for APP, BACE1, GR, GPx1, pGSK-3β, BDNF, and α-tubulin were obtained from Santa Cruz (Dallas, TX, USA). Antibodies against pAkt and pPI3K were purchased from Cell Signaling Technology (Danvers, MA, USA).

### 4.2. Animals and Experimental Design

Male ICR mice (5 weeks old) were purchased from Orient Bio Co. (Gyeonggi, Korea) and maintained in the animal facility located at CHA University (Gyeonggi, Korea). The project was approved on 5 January 2017, by the Institutional Animal Care and Use committee of CHA University (IACUC approval number: 170016). Mice were individually housed for 1 week under a 12 h light/dark cycle at a temperature of 20–24 °C and humidity of 44.5–51.8%. After 1 week of adaptation, mice were randomized into four groups (six per group). The mice were orally administered SM70EE (150, 450 mg/kg/day) or an equal volume of vehicle 1.0% methylcellulose once daily for 2 weeks. After 2 weeks, mice received I.C.V. injections of Aβ_1–42_ (400 pmol). After the Aβ_1–42_ injection, the mice were orally administered SM70EE for an additional 2 weeks (as described above). The behavioral tests were conducted after completion of the oral SM70EE administration.

### 4.3. I.C.V. Injection of Aβ_1–42_

Mice (*n* = 6) were anesthetized with 1.2% tribromoethanol (Avertin), and the stereotaxic surgical procedure was conducted using a stereotaxic surgical frame with a mouse adaptor (David Kopf Instruments, Tujunga, CA, USA). The Aβ_1–42_ (400 pmol, 5 μL) was administered I.C.V. using a Hamilton microsyringe (−1.5 mm anteroposterior, 1 mm mediolateral, and 2 mm dorsoventral tobregma).

### 4.4. Passive Avoidance Test

The passive avoidance test was conducted as previously described [44]. Briefly, mice were tested in a passive avoidance shuttle box (Gemini, San Diego, CA, USA) that was divided into two chambers (illuminated and dark). On the first day, mice performed a training trial in the shuttle box. Each mouse was placed in the illuminated compartment for 2 min. When the mouse entered the dark compartment, the door was closed, and the mouse received an electric foot shock (0.5 mA, 2 s) through the floor. After 24 h, the test was repeated. The mouse was placed in the illuminated compartment, and the time to enter the dark compartment was recorded (maximum time of the step-through latency was 300 s).

### 4.5. Morris Water Maze Test

The Morris water maze test was conducted as previously described [45]. Briefly, the water maze consisted of a circular pool (90 cm in diameter) filled with water to 30 cm and maintained at 20 ± 1 °C. The area of the maze was virtually divided into four equal quadrants. A white escape platform was placed in the center of one quadrant and submerged 1 cm below the surface of the water. Swimming behavior was monitored and analyzed by a Smart video camera (version, 2.5.21; Panlab, Cornellà, Spain) connected to a video-tracking system. Behavior was examined for three trials per day for 4 days. The time required to find the platform was recorded to determine the memory processing ability in the mice (maximum escape latency during the test was 60 s).

### 4.6. Biochemical Analysis

Hippocampal tissue was homogenized with PBS and centrifuged at 12,000× *g* for 20 min at 4 °C to obtain the supernatant, which was used to determine the levels of Aβ_1–42_, GSH, BDNF, and AChE.

To determine the level of Aβ_1–42_, the supernatant was added to 96-well plates and incubated for 90 min at 37 °C. After incubation, the supernatant was removed and biotinylated antibodies were added to the plates. The wells were incubated for 1 h at 37 °C. The antibody was aspirated, and the plate was washed three times. Horseradish peroxidase conjugate was added and incubated for 30 min at 37 °C. The plates were washed five times. After washing, a substrate reagent was added and incubated for 15 min at 37 °C. Absorbance was determined using the ELISA plate reader PowerWave HT (BioTek, Winooski, VT, USA) at a wavelength of 450 nm.

To determine the level of GSH, supernatants were added to 96-well plates. Assay cocktails were added and incubated for 25 min at 37 °C. After incubation, absorbance was determined using the PowerWave HT ELISA plate reader at a wavelength of 410 nm.

To examine the level of BDNF, supernatants were added to 96-well plates and incubated for 90 min at 37 °C. After incubation, supernatants were discarded and biotinylated anti-mouse BDNF antibody was added to the plates. The plates were incubated for 1 h at 37 °C. Then, the plates were washed three times with 0.01 M PBS. The ABC working solution was added and incubated for 30 min at 37 °C. The plates were washed five times. After washing, the 3,3′,5,5′-tetramethylbenzidine color-developing agent was added and incubated in the dark for 25–30 min at 37 °C. Absorbance was determined using the PowerWave HT plate reader at a wavelength of 450 nm.

To measure the level of AChE, supernatants were added to 96-well plates. The AChE reaction mixtures were then added and incubated for 10 min at 37 °C. After incubation, absorbance was determined using the PowerWave HT plate reader at a wavelength of 410 nm.

### 4.7. Western Blot Analysis

For protein extraction, hippocampal tissues were homogenized with lysis buffer (RIPA lysis and extraction buffer, Thermo Fisher Scientific, San Jose, CA, USA) containing protease with phosphatase inhibitor cocktails 2 and 3.The lysates were clarified by centrifugation at 12,000× *g* for 20 min at 4 °C. Protein samples (20 µg) were separated by sodium dodecyl sulfate polyacrylamide gel electrophoresis and transferred onto polyvinylidene fluoride membranes. The membranes were blocked with 5% skim milk and immunoblotted with primary antibodies specific for the indicated proteins overnight. Secondary antibodies conjugated with horseradish peroxidase (1:5000) were applied for 4 h. The bands were visualized by enhanced chemiluminescence, and proteins were detected with LAS image software (Fuji, Valhalla, NY, USA).

### 4.8. Statistical Analysis

All statistical analyses were performed using the Statistical Package for Social Sciences version 12.0 (Armonk, NY, USA). A one-way analysis of variance (ANOVA) was used for comparisons among groups. Significant differences between the mean values were assessed using Duncan’s test. The *p*-values in the multiple comparison results (e.g., a, b, c, and d) indicated significant differences among the groups (*p* < 0.05).

## 5. Conclusions

Our results showed that SM70EE ameliorated cognitive impairments and attenuated the oxidative stress induced by I.C.V. injection of Aβ_1–42_ in mice. These findings indicated that SM70EE-mediated inhibition of GSK-3β phosphorylation may be regulated by the BDNF/PI3K/Akt signaling pathway and thereby may suppress the activation of APP processing caused by I.C.V. injection of Aβ_1–42_ in mice. We speculate that SM70EE administered together with other therapeutics may induce synergistic effects to ameliorate cognitive dysfunction.
